# Modified Anderson-Darling Test-Based Target Detector in Non-Homogenous Environments

**DOI:** 10.3390/s140916046

**Published:** 2014-08-29

**Authors:** Yang Li, Yinsheng Wei, Bingfei Li, Gil Alterovitz

**Affiliations:** 1 Department of Electronic Engineering, Harbin Institute of Technology, Harbin 150001, China; E-Mails: weiys@hit.edu.cn (Y.W.); westerange@163.com (B.L.); 2 Center for Biomedical Informatics, Harvard University, Boston, MA 02115, USA; E-Mail: gil_alterovitz@hms.harvard.edu

**Keywords:** target detection, Constant False Alarm Rate (CFAR) detector, Anderson-Darling (AD) test, statistical signal processing, clutter edge, non-homogenous background

## Abstract

A constant false alarm rate (CFAR) target detector in non-homogenous backgrounds is proposed. Based on K-sample Anderson-Darling (AD) tests, the method re-arranges the reference cells by merging homogenous sub-blocks surrounding the cell under test (CUT) into a new reference window to estimate the background statistics. Double partition test, clutter edge refinement and outlier elimination are used as an anti-clutter processor in the proposed Modified AD (MAD) detector. Simulation results show that the proposed MAD test based detector outperforms cell-averaging (CA) CFAR, greatest of (GO) CFAR, smallest of (SO) CFAR, order-statistic (OS) CFAR, variability index (VI) CFAR, and CUT inclusive (CI) CFAR in most non-homogenous situations.

## 1. Introduction

Most target Constant False Alarm Rate (CFAR) detection algorithms are designed for a particular family of clutter distribution models. However, echo data in real environments are usually non-homogeneous and do not follow the assumed probability distribution model independent of which remote sensors are used such as radar, sonar, or chemical-detection sensors [1,2]. This is due to the multi-source detection environment, which degrades detection performance especially in multi-target and clutter edge backgrounds [3].

The earliest CFAR detector, Cell Average (CA)-CFAR [4], is optimal in a homogeneous background when the reference cells follow an independent and identical distribution (IID) to the cell under test (CUT) by an exponential distribution. It does, however, suffer a serious performance degradation in multi-target and clutter edge backgrounds [5]. Later modifications of CA-CFAR include the Greater Of (GO) CFAR, which can minimize the false alarm rate in the case of a clutter edge, [6] and the Smaller Of (SO) CFAR, which offers better performance in a multiple target environment [7].

Approaches to improve performance in both homogenous and non-homogenous detection backgrounds fall into two broad categories [8]. One focuses on the modifications of the CA-CFAR, such as ACCA-CFAR [9]. The other detects the presence of non-homogeneity in the CFAR window before applying suitable CFAR methods. One example of the latter is Variability Index (VI)-CFAR, which provides low CFAR loss in a homogeneous environment and performs robustly with multiple targets and clutter edges [10]. However, the performance of the VI detector degrades when interfering targets are not confined to one side of the Cell Under Test (CUT). Multiple possible modifications to the VI-CFAR detector are used to improve the performance in such cases. Two examples are IVI-CFAR [11], MVI-CFAR [12].

Thus, it can be concluded that identifying the type of the background may be the key to optimizing the detector in a multi-target or clutter edge environment. The current literature shows that clutter classification can improve the performance of detectors. Bouvier [13] used a statistical distribution model for clutter identification to classify the detection background into one of three categories: sea clutter, ground clutter or compound clutter. Oliver [14] introduced a method that uses textural features as the attributes for clutter representation. The class selection is determined by fitting parameters to the statistical distribution.

In recent years the research community has proposed several intelligent method based solutions. Neinavaie [15] proposes a preprocessing algorithm, which can classify clutter using an on-line intelligent method. This method obtains a radar clutter map without geodata of the environment. Li proposes a cognitive detector that uses statistical distribution and image features to recognize clutter on-line [16]. The multi-strategy detector makes a decision based on the various parameters of the probability distribution function for each particular background. Pierucci [17] introduces a knowledge-based detection system that utilizes 11 feature values for recognizing types of clutter. These are derived from the second moments about the origin as the attributes of echoes.

Another problem with the current methodology is that real datasets do not necessarily follow the assumed identical distribution of a prototypical clutter series. Testing the homogeneity of samples before identifying the distribution model for sample series may be a way to solve this problem. Zhang [18] proposed the AD-CFAR detection based on a K-sample AD test and analyzed its performance in a Rayleigh background. Kim [19,20] introduced an AD test based CUT inclusive (CI) CFAR algorithm using a 3 × 3 two-dimensional reference window as the minimum reference block. Better performance can be achieved by accumulating more reference cells.

This paper proposes a more effective method for clutter classification based on modified K-sample Anderson-Darling (AD) tests. Double partition test, clutter edge refinement and outlier eliminating are incorporated into the algorithm in order to improve performance in multi-target and clutter edge backgrounds. Comparison between different algorithms is based on the same number of reference windows. In the 2nd section of the paper, the basic theory of K-sample AD tests is introduced, and a homogenous clutter block extraction method based on Modified AD (MAD) tests is proposed. Comparison and simulation results are presented in Section 3 under homogenous, multi-target and clutter edge backgrounds. Conclusions drawn from these results are presented in Section 4.

## 2. Homogenous Clutter Extraction Based on Modified AD Test

### 2.1. K-Sample AD Tests

K-sample AD tests [21] are used for testing the homogeneity of samples when the clutter model is unknown.

We test the hypothesis:

(1) $$H_{0}:F_{1}=F_{2}=\cdots =F_{K}$$

where *F_1_*, *F_2_*,*…*, *F_K_* are the Cumulative Distributed Functions (CDF) of clutter block. Let the clutter block vector be defined as:

(2) $$X=[B_{1},B_{2},\cdots ,B_{K}]$$

(3) $$B_{i}=\{x_{1},x_{2},\cdots ,x_{n_{i}}\}$$

where *n_i_*, is the number of the samples in block *B_i_*, *N* = Σ*n_i_* and *F_In_i__* is the Empirical Distributed Function (EDF) of block *B_i_*. The test statistics of K-sample AD is:

(4) $$A_{K}^{2}=\sum_{i=1}^{K}n_{i}\int_{D_{N}}\frac{{\left[F_{In_{i}}(x)-H_{N}(x)\right]}^{2}}{H_{N}(x)[1-H_{N}(x)]}dH_{N}(x)$$

where *H_N_*(*x*) is the EDF of *N* clutter samples and *D_N_* = {*x* ∈ *R*:*H_N_* (*x*) <1}.

Let *Z*_1_, *Z*_2_,…, *Z_N_* be the ascending sequence of *x*_1_, *x*_2_,…*x_ni_*. Thus *M_ij_* denotes the number of the clutter samples in *B_i_*(1 ≤ *i* ≤ *K*) which are no larger than *Z_j_*(1≤ *j* ≤ *N*). Then $A_{KN}^{2}$ can be computed as:

(5) $$A_{KN}^{2}=\frac{1}{N}\sum_{i=1}^{K}\frac{1}{n_{i}}\sum_{j=1}^{N-1}\frac{{\left(NM_{ij}-jn_{i}\right)}^{2}}{j(N-j)}$$

The expected outcome and variance of the test statistic $A_{KN}^{2}$ are:

(6) $$E[A_{KN}^{2}]\approx K-1$$

(7) $$\sigma_{N}^{2}=\mathrm{var}(A_{KN}^{2})=\frac{aN^{3}+bN^{2}+cN+d}{\left(N-1)(N-2)(N-3\right)}$$

with coefficients of:

(8) $$\begin{array}{l} a=(4g-6)(K-1)+(10-6g)H\\ b=(2g-4)K^{2}+8hK+(2g-14h-4)H-8h+4g-6\\ c=(6h+2g-2)K^{2}+(4h-4g+6)K+(2h-6)H+4h\\ d=(2h+6)K^{2}-4hK \end{array}$$

where:

(9) $$\begin{array}{l} H=\overset{K}{\underset{i=1}{\text{∑}}}\frac{1}{n_{i}}\\ h=\overset{N-1}{\underset{i=1}{\text{∑}}}\frac{1}{i}\\ g=\overset{N-2}{\underset{i=1}{\text{∑}}}\overset{N-1}{\underset{j=i+1}{\text{∑}}}\frac{1}{(N-i)j} \end{array}$$

It was previously shown that a homogeneity test of *H*_0_ may be carried out by comparing the degree of approximation between $A_{KN}^{2}$ and a Gaussian distribution [18]. The statistic *T_KN_* after $A_{KN}^{2}$ is normalized as:

(10) $$T_{KN}=\frac{A_{KN}^{2}-E[A_{KN}^{2}]}{\sigma_{N}}\approx \frac{A_{KN}^{2}-(K-1)}{\sigma_{N}}$$

If *T_KN_* < *t_K_*_−1_(*α*), then *H*_0_ is not rejected. Here, *t_K_*_−1_(*α*) is the critical threshold of *T_KN_* with the confidence level of *α*. If *m* = *K* − 1, then:

(11) $$t_{m}(\alpha )=b_{0}+\frac{b_{1}}{\sqrt{m}}+\frac{b_{2}}{m}$$

with *b*_0_, *b*_1_, *b*_2_ derived in previous work [21].

### 2.2. Homogeneous Clutter Block Extraction

The detection probability *P_d_* and the false alarm probability *P_f_* are derived based on ideal detection conditions in a nonhomogeneous environment, *i.e.*, the clutter block *B_i_*(1 ≤ *i* ≤ *K*) can be detected when there are interfering targets and clutter edge in the background [18,20].

**Figure 1.**
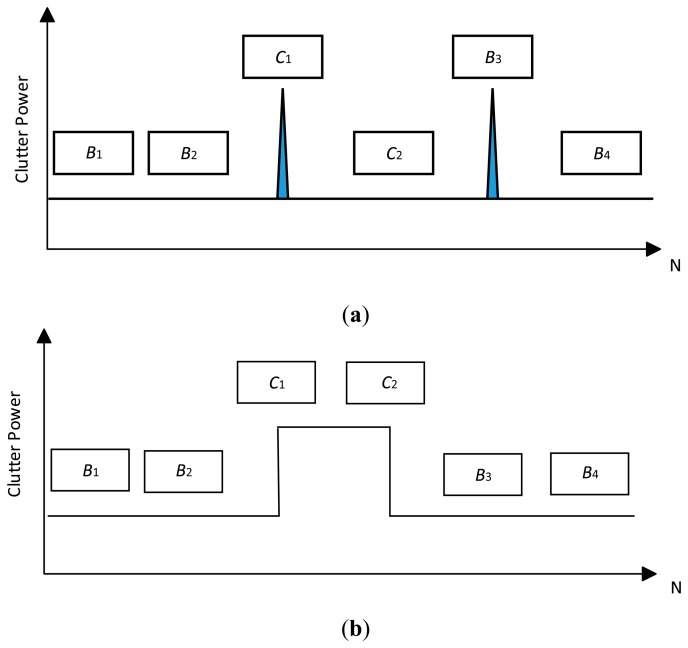
Sketch of non-homogenous background (**a**) The background with interferences; (**b**) The background with straddling clutter.

**Figure 2.**
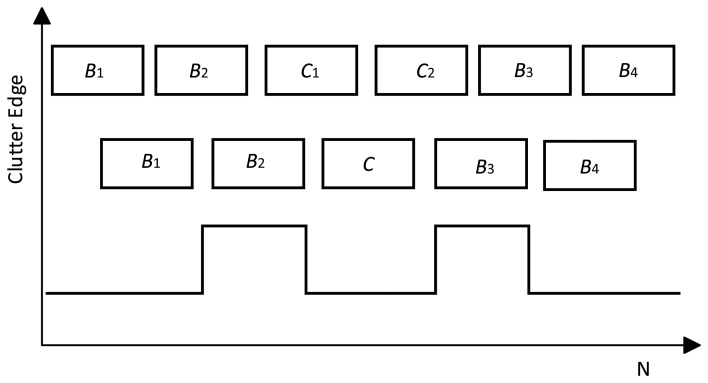
Double partition test.

However, such circumstances cannot be assumed (see Figure 1). For example, in AD tests, let the sample numbers of the clutter blocks *B*_1_ and *B*_2_ be six, and block *B*_1_ be homogeneous. These two clutter blocks can be identified as homogenous with a confidence level *α* according to Equations (10) and (11). Thus, in this situation, a Modified AD (MAD) CFAR detector is proposed in order to improve the performance. Compared with the AD-CFAR detector, a MAD based CFAR detector includes the operations of double partition test, clutter edge refinement and outlier eliminating as follows:

The procedure for the double partition test is shown in Figure 2. Let the reference window be divided into six clutter sub-blocks of equal length. Initially, the 6-sample AD test is performed on the entire reference window. The normalized $A_{KN}^{2}$, *i.e.*, *T_KN_*, being less than *t_K_*_−1_(*α*), does not automatically fail to reject *H*_0_ because the K-sample AD test cannot delete the nonhomogeneous clutter sub-block shown in Figure 1 effectively. Thus, the double partition test for the whole reference window is necessary after calculating the 6-sample AD test statistic *T_KN_* < *t_K_*_−1_(*α*). In this step, five equally spaced clutter blocks are generated by localizing the clutter block edge to the center of the clutter block in the 1st partition test. This 5-sample AD test may fail to reject *H*_0_ if *T_KN_* < *t_K_*_−1_(*α*). As non-homogenous clutter are shown in Figure 1b, high power clutter will fill in a clutter sub-block during the 2nd partition test if it straddles between two clutter blocks. In this case, a K-sample AD test can identify the nonhomogeneous reference window effectively, and then use the subsequent homogeneity identification method for the clutter sub-block.

**Figure 3.**
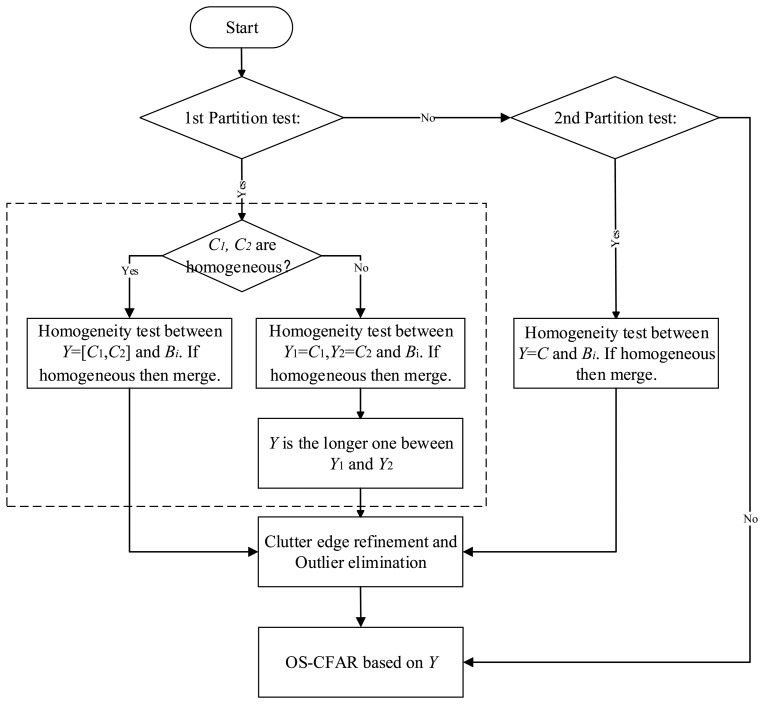
Flow chart of homogenous clutter block extraction.

After the double partition tests, a 2-sample AD test will work on the sub-blocks for homogeneous clutter block extraction and merging in the case that *H*_0_ is rejected. The simplified flow chart is shown in Figure 3. The procedures in the block with dotted line in Figure 3 are:

Step 1 Divide the reference samples *x*_1_, *x*_2_,…, *x*_n_ into *d* = [*n*/*m*] blocks, each block has *m* samples.
Step 2 Let the clutter block next to the CUT be *C*_1_, *C*_2_, others be *B*_1_, *B*_2_,…, *B*_d−2_, and *Y* = {*C*_1_, *C*_2_}.
Step 3 K-sample AD test works on *C*_1_, *C*_2_ (*K* = 2), if they are homogenous, then go to Step 4, otherwise test whether *B_i_* (*i* = 1, 2,…, *d* − 2) is homogenous as *C*_1_, *C*_2_. Merge homogenous blocks and get *Y_1_* = {*C*_1_, *B_i_*,}, *Y_2_* = {*C*_2_, *B_i_*,} till *i* = *d* − 2. Select the longer one between *Y_1_* and *Y_2_*, and let it be *Y*.
Step 4 Test whether *Y* and *B_i_* (*i* = 1, 2,…, *d* − 2) are homogenous. If yes, then *Y* = {*C*_1_, *C*_2_, *B_i_*}, till *i* = *d* − 2.

**Figure 4.**
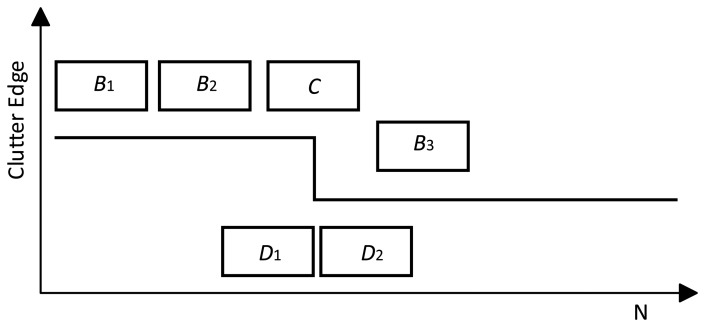
Clutter edge refinement.

Clutter edge refinement is shown in detail in Figure 4. The algorithm described above will identify block *C* as it has the same distribution as *B*_1_ and *B*_2_ with a high probability if the clutter block *C* consists of clutter edge as is shown in Figure 4. In the clutter edge refinement section, a 2-sample AD test is operated on *D*_1_ and *D*_2_ to locate the new position of the clutter edge to see if *D*_1_ and *D*_2_ have the same distribution. If yes, *C* can be determined to have the same distribution as *B*_1_ and *B*_2_. If no, the first part of *C* is identified as the same distribution as *B*_1_ and *B*_2_. Thus the accuracy of the extraction is a half-length of the sub-block, which can improve performance in clutter edge environment.

To eliminate the effect of multi-interference, an outlier-eliminating operation should follow the clutter homogeneity extraction. A conventional and effective curvilinear regression analysis [22] method is applied to the reference window. The upper confidence bound value can be set as 100(1−*α*). Then 2 or 3 order unary non-linear regression curves can be generated to estimate the background level of CUT to eliminate the outliers based on the least square method.

## 3. Performance Comparison and Analysis

In this section, Monte Carlo simulations were performed to test the performance of the MAD-CFAR detector in homogeneous, multi-target and clutter edge environments. The length of the reference window *N* is 36 for MAD-CFAR and CI-CFAR, which can be divided into 6 blocks for MAD-CFAR processing. The confidence level is 0.01 for AD test. For CI-CFAR, the 3 × 3 sub-block is resized to 6 × 1 sub-block. The compared CFAR algorithms are CA-CFAR, GO-CFAR, SO-CFAR, VI-CFAR, CI-CFAR, OS-CFAR. For VI-CFAR, *K_vi_* is 6.72, and *K_mr_* is 2.064. For OS-CFAR, *k* is 3 × *N*/4 where *N* is the number of reference cells. The noise of the detection background obeys exponential distribution. The upper confidence bound value of the outlier-elimination method is set at 0.85 (*α*=0.15). Table 1 describes the target and clutter scenario of each simulation and summarizes the simulation results.

### 3.1. Homogeneous Environment

**Figure 5.**
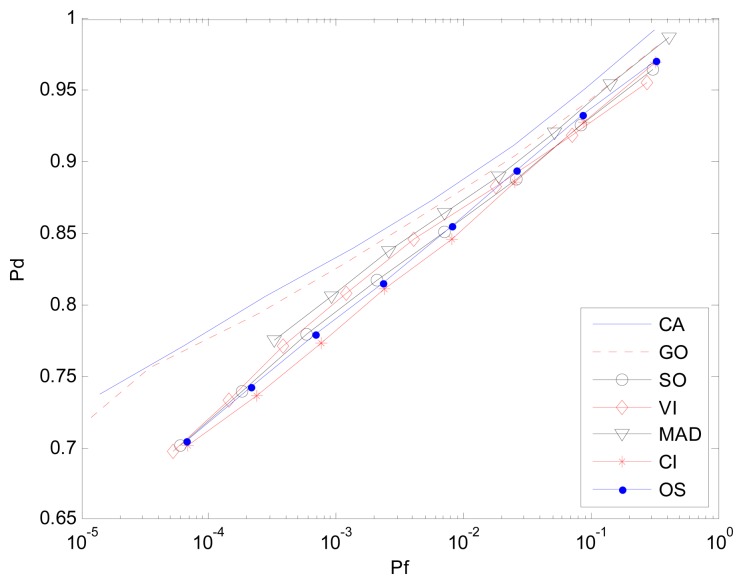
*P_d_ vs. P_f_* (ROC) in homogeneous environment.

In the homogeneous environment, all the clutter sub-blocks follow the same distribution. For K-sample AD test, the MAD-CFAR detector can determine whether or not the selected clutter blocks are homogeneous with a high degree of certainty. For the Receiver Operation Characteristic (ROC) curve (*P_d_ vs. P_f_*) *P_f_* is set from 10^−4^ to 10^−0.5^, Signal-Noise-Ratio (SNR) of the target in CUT is 15 dB and the number of Monte Carlo simulation times is 10^6^. Figure 5 represents the case in which all of the background clutter is homogenous. To test the detection performance of different CFAR algorithms, the number of reference windows is set to be identical. The ROC curves of MAD, GO, CA, VI-CFAR detector nearly coincide. However, the SO-CFAR and CI-CFAR detector show the worst performance, as is expected in [20] and [23] with the same number of reference cells as other detectors.

### 3.2. Multi-Target Environment

**Figure 6.**
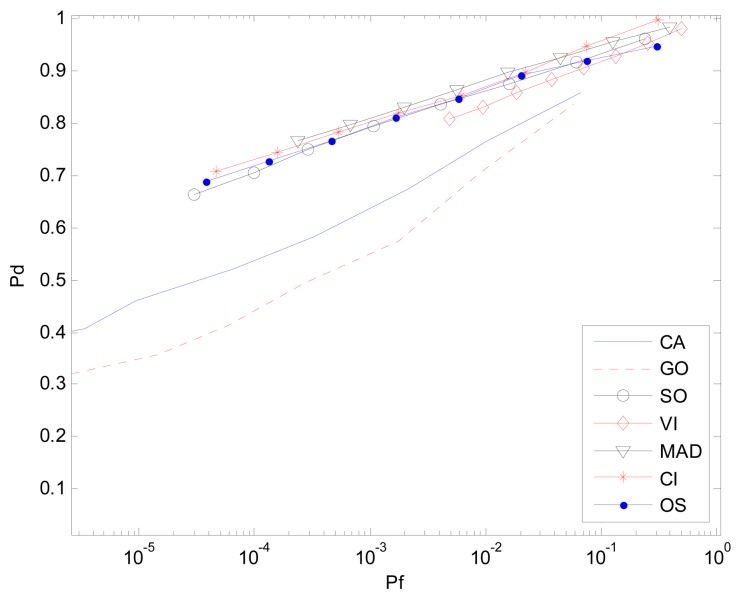
*P_d_ vs. P_f_* (ROC) in the non-homogenous environment with one interference, r(0,1).

In the multi-target background, an AD based clutter extraction algorithm (CI-CFAR) cannot eliminate the clutter block with interference effectively as is shown in Figure 1a. Similar to the CA-CFAR method, it will determine whether or not the current clutter block follows the same distribution and select the whole reference window to estimate the power level of the clutter. Figure 6 is the result of the environment given one interference. Here r(0,1) means one interference is located on one half of the CUT whereas r(1,1) represents one interference is located on each half of the CUT. The Clutter-Noise-Ratio (CNR) or Interference-Noise-Ratio (INR) is 20 dB and SNR is 15 dB. *P_f_* and simulation time are same as in Section 3.1.

**Figure 7.**
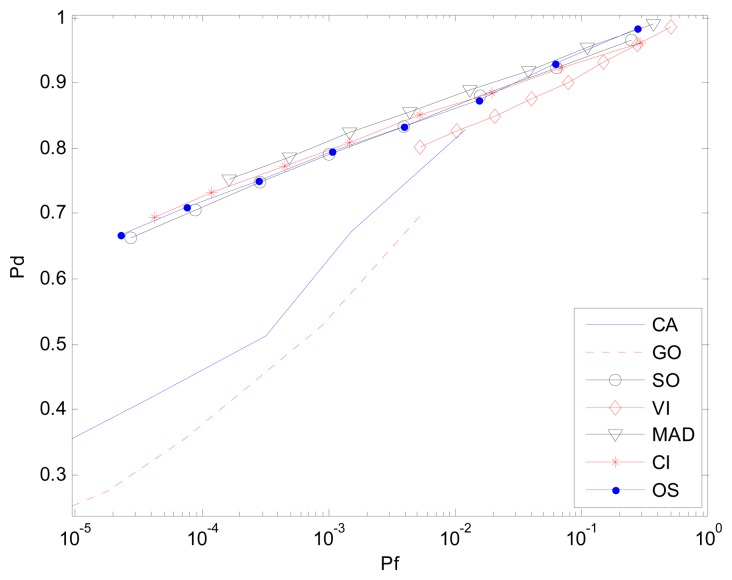
*P_d_ vs. P_f_* (ROC) in the non-homogenous environment with two interferences in one half of the reference window, r(0,2).

**Figure 8.**
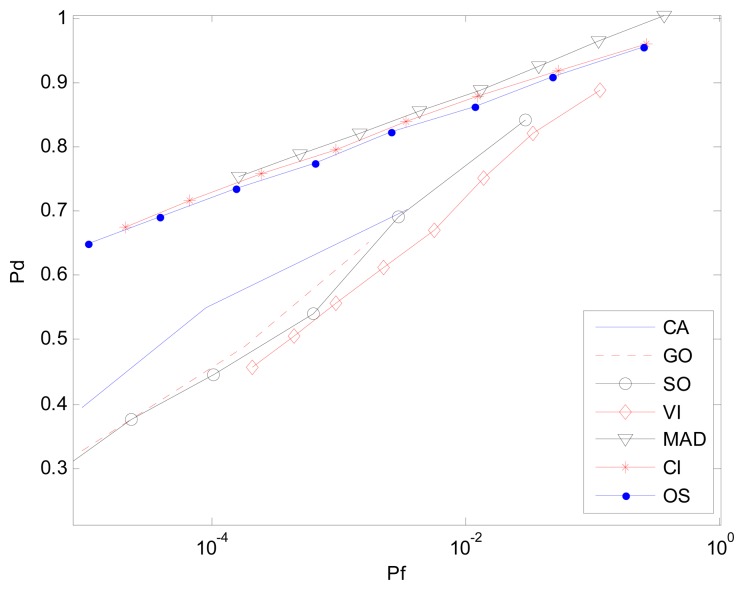
*P_d_ vs. P_f_* (ROC) in the non-homogenous environment with one interference in each half of the reference window, r(1,1).

Figures 7 and 8 are simulation results for the multi-interference environment with two interferences. Both INRs are 20 dB and SNR is 15 dB. Figure 7 is the case where there are two interferences on one half side of CUT. The ROC curves show nearly the same result as is in Figure 6. It indicates that the number of interferences on one half side of CUT does not affect the detection performance too much. However, Figure 8 with r(1,1) shows a different scenario especially for SO, GO and VI, which are sensitive to homogenous/non-homogenous of one half side of CUT.

Considering the MAD-CFAR method, outlier elimination in the extracted homogeneous clutter sequence can help improve the performance in a multi-interference environment. Although K-sample cannot eliminate clutter sub-blocks with multiple targets effectively, outlier eliminating can act as an anti-interference method. It deletes the maximum value sample and uses the other values as references to estimate the clutter power in the clutter sub-block. It can thus increase the detection probability while maintaining the false alarm rate. As is shown in Figures 6, 7 and 8, the proposed MAD based detector has the best performance, particularly when the number of interference targets is greater than one. CA-CFAR, VI-CFAR, alternatively, is sensitive to the scenarios where interference is located on one half or both half sides of CUT.

**Figure 9.**
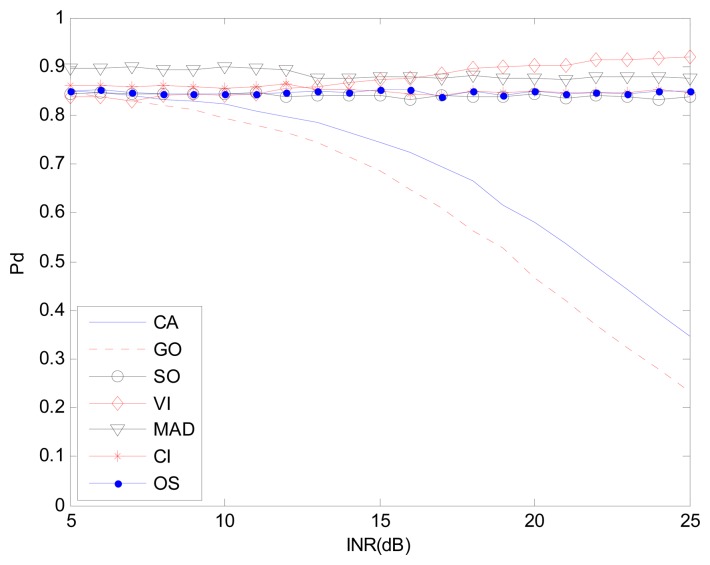
*P_d_ vs.* INR in the non-homogenous environment, r(0,1).

**Figure 10.**
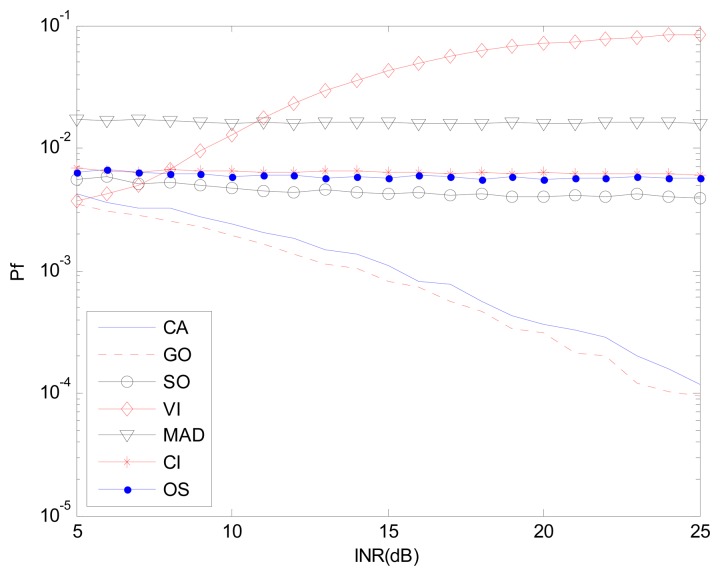
*P_f_ vs.* INR in the non-homogenous environment, r(0,1).

Figures 9 and 10 correspond to the non-homogenous environment with r(0,1), with *P_d_ vs.* INR and *P_f_ vs.* INR plots. These help explain *P_d_* control or CFAR control capabilities of detectors. The INR is set from 5 dB to 25 dB, SNR is 15 dB, *P_f_* is 10^−2^, and the number of simulation times is 10^4^.

**Figure 11.**
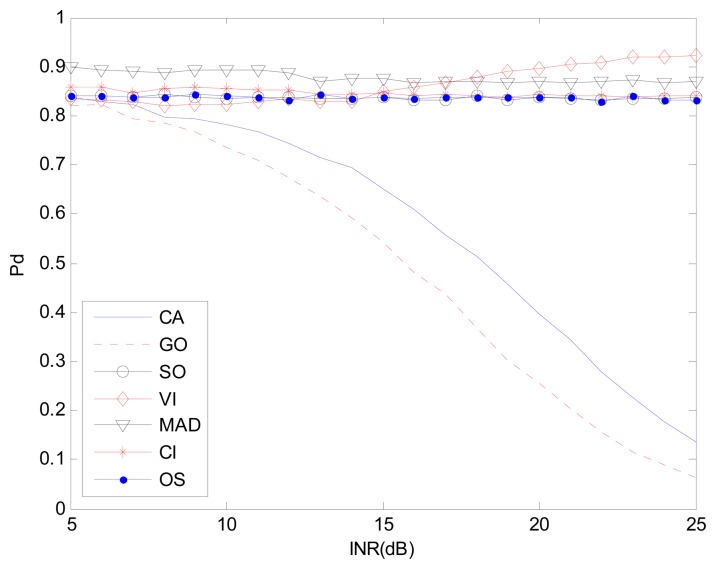
*P_d_ vs.* INR in the non-homogenous environment, r(0,2).

**Figure 12.**
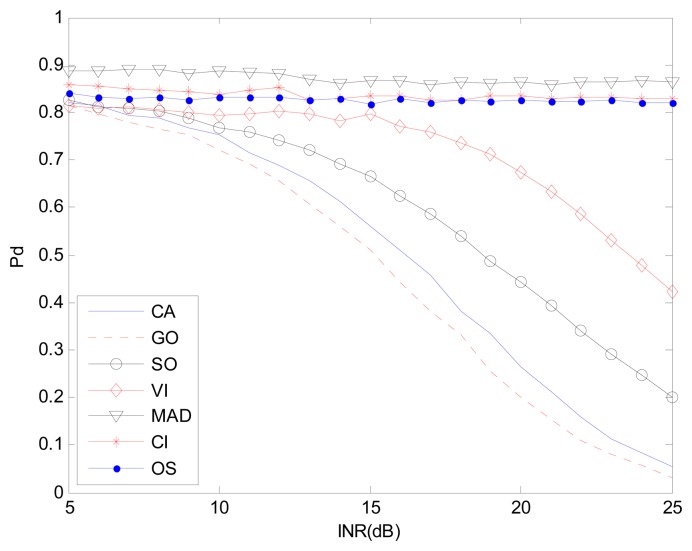
*P_d_ vs.* INR in the non-homogenous environment, r(1,1).

Figures 9, 11 and 12 correspond to the non-homogenous environments with r(0,1), r(0,2) and r(1,1) respectively, and show how MAD-CFAR can control CFAR according to the change of INR. Smoother is better along the INR axis in these figures.

**Figure 13.**
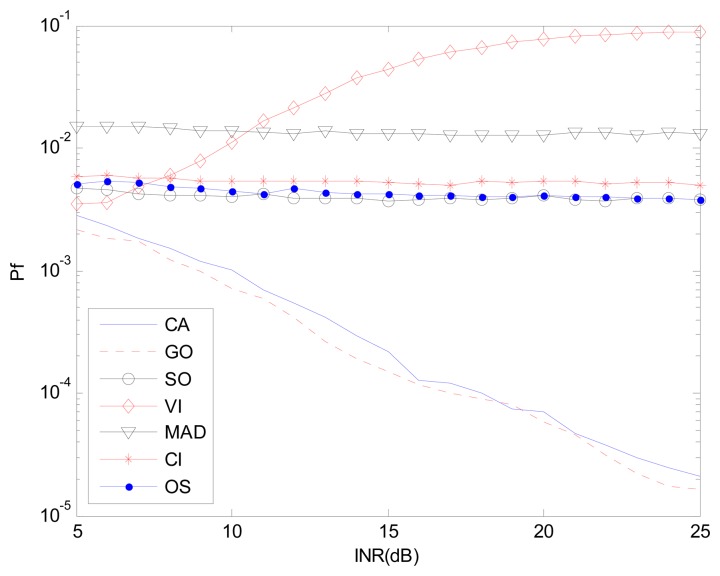
*P_f_ vs.* INR in the non-homogenous environment, r(0,2).

**Figure 14.**
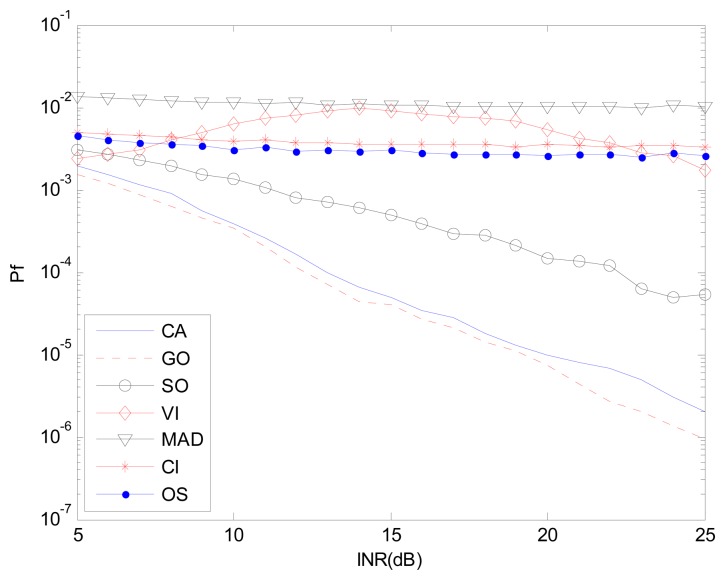
*P_f_ vs.* INR in the non-homogenous environment, r(1,1).

Figures 10, 13 and 14 are simulation results with *P_f_ vs.* INR to show the *P_f_* control capability with different types of interference. It can be concluded that in the case of r(1,1), MAD-CFAR outperforms other detectors in ROC, CFAR control or *P_f_* control capabilities. For the case of r(0,1) and r(0,2), MAD is one of the best detectors, with better ROC and CFAR control performance.

### 3.3. Clutter Edge Environment

When the clutter edge crossed CUT, the *P_f_* degraded sharply especially when the number of reference cells occupied by the clutter edge is equal to half of the number of the reference window. Let CNR be 10, SNR be 25, and *N_c_* be *N*/2, which is the number of reference cells occupied by the clutter edge. Other simulation parameters are the same as in Section 3.1.

**Figure 15.**
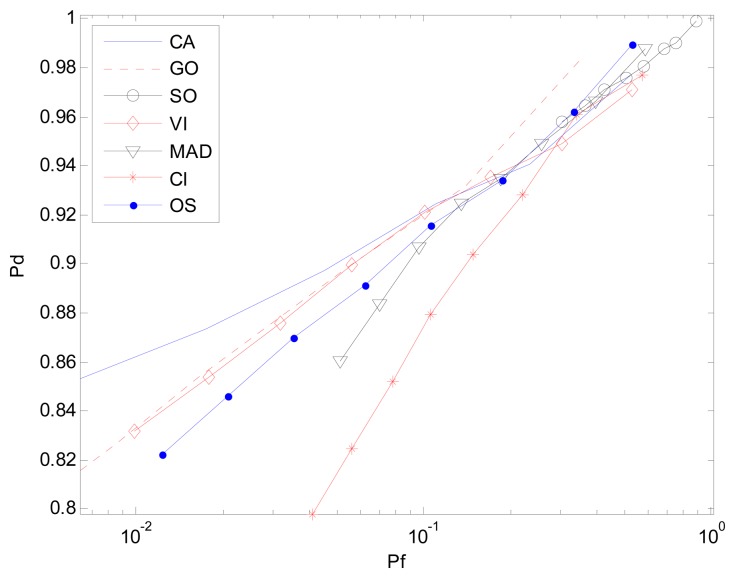
*P_f_ vs. P_d_* (ROC) in clutter edge environment.

Figure 15 is the result of the case in clutter edge with the above-mentioned simulation parameters. By using an edge refinement method, MAD-CFAR outperforms CI-CFAR and is close to SO-CFAR.

**Figure 16.**
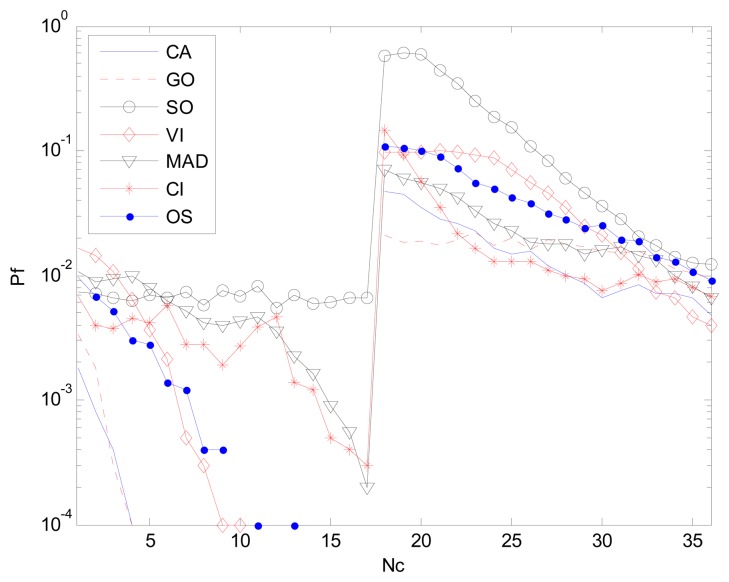
The false alarm performance in clutter edge environment.

Figure 16 shows *P_f_ vs. N_c_* where *N_c_* is from 1 to 36, *P_f_* is 10^−2^, and the number of simulation times is 10^4^. Other simulation parameters are same as in Section 3.1. The peaks crossing the CUT is the significant sign showing the false alarm control capability in clutter edge. A higher peak represents worse false alarm control capability. MAD-CFAR outperforms VI-CFAR, OS-CFAR, CI-CFAR and SO-CFAR in this measure. It had an inferior performance when compared to GO-CFAR and CA-CFAR. When *N_c_* is bigger than *N*/2, the smoothness along *N_c_* axis can show the CFAR control capabilities of detectors. In this case, MAD-CFAR outperforms OS-CFAR, CI-CFAR, VI-CFAR and SO-CFAR.

We can conclude that using the two-time reference window partition, clutter edge refinement method help the proposed MAD-CFAR detector outperforms the CI-CFAR in ROC and have a good CFAR control capability.

### 3.4. Computation Cost

Table 2 lists the computation cost with 1000 times Monte Carlo simulation with Intel Core i7-2600 CPU @ 3.4 GHz, 4 GB memory. MAD-CFAR costs a little more (1.16 times) than CI-CFAR.

## 4. Conclusions

A modified AD test-based (MAD) CFAR algorithm is proposed in this paper. K-sample AD tests, double partition tests, clutter edge refinement and outlier elimination are used for target detection in a non-homogenous environment, such as multi-interference and clutter edge background. Simulations show that the proposed method has a perfect CFAR control capability and high detection performance in the background with multi-interferences and clutter edge. The results indicate that the MAD-CFAR detector outperforms CA-CFAR, GO-CFAR, SO-CFAR, OS-CFAR, VI-CFAR, CI-CFAR in most situations.

## Figures and Tables

**Table 1.** Summary of results.

Type of the Environments	Performance (A > B Means A is Better than B, A ≈ B Means A is Almost Same as B)
Homogenous	(*P_d_ vs. P_f_*): CA>GO>**MAD**>VI>SO> OS>CI

One interference r(0,1)	*P_d_ vs. P_f_* (ROC): **MAD**>CI>OS>SO>VI>CA>GO
	CFAR control(INR *vs. P_f_*): **MAD**≈SO≈CI≈OS>VI>CA>GO
	*P_d_* control(INR *vs. P_d_*): SO≈CI≈OS>**MAD**≈VI>CA>GO

Two inteferences on one half side of the CUT r(0,2)	*P_d_ vs. P_f_* (ROC): **MAD**>CI>OS>SO>VI>CA>GO
	CFAR control(INR *vs. P_f_*): **MAD**≈SO≈CI≈OS>VI>CA>GO
	*P_d_* control(INR *vs. P_d_*): SO≈CI≈OS>**MAD**≈VI>CA>GO

One inteferences on each half side of the CUT r(1,1)	*P_d_ vs. P_f_* (ROC): **MAD**>CI>OS> CA>GO> SO>VI
	CFAR control(INR *vs. P_f_*: **MAD**≈CI≈OS>VI>SO>CA>GO
	*P_d_* control(INR *vs. P_d_*): **MAD**≈CI≈OS>VI>SO>CA>GO

Clutter edge	*P_d_ vs. P_f_* (ROC) (*N_c_* = N/2): CA>GO>VI>OS>**MAD**≈SO>CI
	*N_c_ vs. P_f_* (*N_c_* = N/2): GO>CA>**MAD**≫VI>OS>CI>SO
	CFAR control (*N_c_* > N/2): GO>CA>**MAD**>OS>CI>VI>SO

**Table 2.** Computation Cost.

Algorithm	Time Cost (s)	(Time Cost) Divided by (Time Cost with CA-CFAR)
CA-CFAR	2.33	1
GO-CFAR	2.61	1.12
SO-CFAR	2.57	1.10
VI-CFAR	2.34	1.00
MAD-CFAR	3.91	1.68
CI-CFAR	3.37	1.45
OS-CFAR	2.66	1.14
